# Chromatin immunoprecipitation: optimization, quantitative analysis and data normalization

**DOI:** 10.1186/1746-4811-3-11

**Published:** 2007-09-24

**Authors:** Max Haring, Sascha Offermann, Tanja Danker, Ina Horst, Christoph Peterhansel, Maike Stam

**Affiliations:** 1 Swammerdam Institute for Life Sciences, Universiteit van Amsterdam, Kruislaan 318, 1098 SM Amsterdam, The Netherlands; 2 Institute for Biology I, Aachen University, Worringer Weg 1, 52074 Aachen, Germany

## Abstract

**Background:**

Chromatin remodeling, histone modifications and other chromatin-related processes play a crucial role in gene regulation. A very useful technique to study these processes is chromatin immunoprecipitation (ChIP). ChIP is widely used for a few model systems, including *Arabidopsis*, but establishment of the technique for other organisms is still remarkably challenging. Furthermore, quantitative analysis of the precipitated material and normalization of the data is often underestimated, negatively affecting data quality.

**Results:**

We developed a robust ChIP protocol, using maize (*Zea mays*) as a model system, and present a general strategy to systematically optimize this protocol for any type of tissue. We propose endogenous controls for active and for repressed chromatin, and discuss various other controls that are essential for successful ChIP experiments. We experienced that the use of quantitative PCR (QPCR) is crucial for obtaining high quality ChIP data and we explain why. The method of data normalization has a major impact on the quality of ChIP analyses. Therefore, we analyzed different normalization strategies, resulting in a thorough discussion of the advantages and drawbacks of the various approaches.

**Conclusion:**

Here we provide a robust ChIP protocol and strategy to optimize the protocol for any type of tissue; we argue that quantitative real-time PCR (QPCR) is the best method to analyze the precipitates, and present comprehensive insights into data normalization.

## Background

Epigenetic regulation of gene expression is crucial for cell differentiation, and thus essential for normal growth and development of higher eukaryotes. Epigenetic control is an intricate interplay between various molecular mechanisms, e.g. DNA methylation and histone modifications (reviewed in [1-4]). Whereas DNA methylation has been studied in great detail for several decades, the role of histone modifications has only been fully appreciated for about 10 years [5]. Since then the number of papers on new histone modifications and their possible functions has exploded.

**Figure 1:**
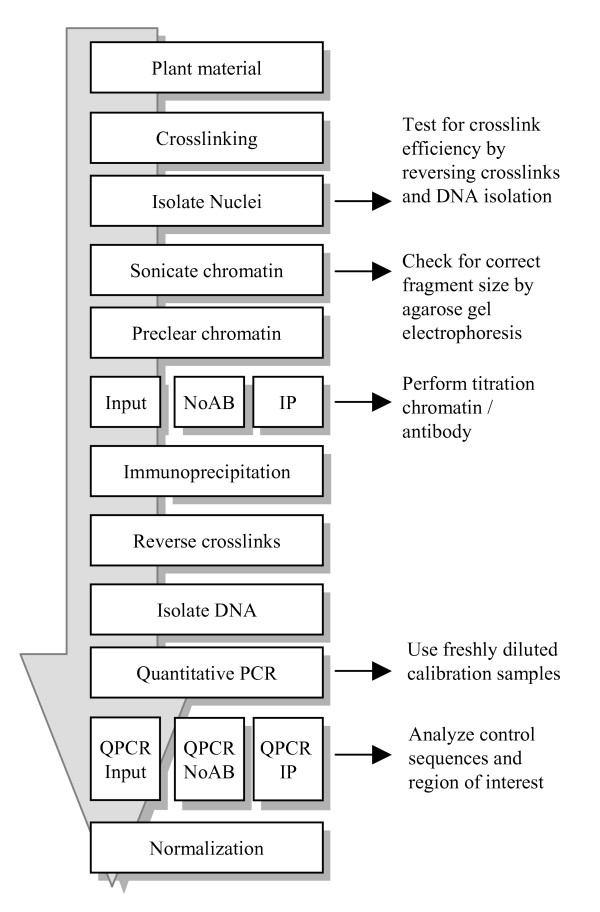
Outline of the ChIP-QPCR procedure. This outline represents the ChIP procedure as described in the text. IP, ChIP sample; NoAb, No-antibody control.

The most widely used procedure to examine histone modifications is Chromatin Immunoprecipitation (ChIP), a technique first established for cultured *Drosophila *cells [6]. In short, ChIP relies on antibodies to identify the presence of specific histone modifications at DNA regions of interest. Chromatin is extracted from cells or tissue, fragmented and incubated with antibodies against specific histone modifications (Figure 1). The chromatin fragments bound to the antibodies are captured using protein A/G beads, and DNA is isolated from the precipitate. This DNA is usually analyzed by (quantitative) PCR to determine the abundance of a region of interest in the precipitated material. ChIP has proven to yield very valuable information on chromatin-associated processes in eukaryotes, including plants and humans. Despite the fact that ChIP on plant material is established for the widely used model system *Arabidopsis*, to implement and optimize the technique for other model plant species, such as maize, is still very challenging and time consuming.

In current literature, conventional PCR is mostly used to analyze plant ChIP precipitates. In this method, the intensity of a DNA band on an agarose gel is assumed to reflect the initial abundance of a specific DNA fragment in the precipitate. Instead, the intensity of the band reflects the endpoint of a non-linear PCR reaction. Since a careful quantification of ChIP signals is important for a correct interpretation of the data, we discuss the application of quantitative real-time PCR (QPCR).

Once the QPCR data are obtained, they can be normalized and presented in different ways. All normalization methods have their own advantages and drawbacks. Therefore, making a well-informed choice is important for correct interpretation of the data. For example, the most commonly used methods, '% of input' (%IP) and 'fold enrichment', may obscure the biological meaning of the ChIP signal by relating the signal intensity to an arbitrary amount of chromatin or to background levels, respectively. Besides the inherent disadvantages of the various methods, the wide range of normalization methods that is currently used can hamper the comparison of published data sets [7].

In this paper we present a robust, optimized ChIP protocol, and in addition a strategy to optimize the protocol when dealing with different experimental systems or conditions. Quantitative real-time PCR (QPCR) is presented as best practice to analyze the precipitated material. A quantitative interpretation of ChIP-QPCR data requires normalization, an often under-illuminated aspect of the ChIP-QPCR procedure. Therefore, this paper provides a discussion on the use of various normalization methods, enabling a well-informed choice for a specific normalization method.

## Results and Discussion

Chromatin immunoprecipitation experiments can roughly be divided into two categories. One uses crosslinked chromatin sheared by sonication (X-ChIP), and the other native chromatin digested by nucleases (N-ChIP). Both methods have their own advantages and disadvantages [8]. Our paper provides and discusses a protocol for X-ChIP that involves formaldehyde crosslinking of the chromatin in intact tissue, ensuring the rapid fixation of the existing chromatin structure. Without crosslinking, we systematically failed to obtain significant amounts of precipitate. In this paper, we discuss the whole procedure in four sections: (1) The isolation of good-quality chromatin from plant material, (2) the chromatin immunoprecipitation itself, (3) the analysis of precipitated material by QPCR and (4) data normalization. An outline of the various steps in the whole procedure is shown in Figure 1.

### (1) Isolation of chromatin

#### Starting material

The isolation of plant chromatin needs a plant-specific approach; plant cells are surrounded by a cell wall, and generally whole tissues, rather than uniform cell cultures, are used to isolate chromatin. Plant cells contain large vacuoles, resulting in a relatively low number of nuclei per gram of tissue. In addition, vacuoles are a source of proteolytic activities [9]. We advise the use of healthy, unfrozen plant tissue as starting material for the isolation of chromatin. If possible, use tissue enriched in unexpanded cells. Such tissue will provide the best yield and purity of the isolated chromatin. In case there are doubts if the tissue of interest will yield good-quality chromatin, non-crosslinked nuclei can be isolated followed by micrococcal nuclease digestion. Good-quality chromatin gives rise to a distinct nucleosome ladder [8,10].

#### Crosslinking

Crosslinking of the starting material by formaldehyde is used to ensure that the chromatin structure is preserved during the isolation and ChIP procedure [6,11]. Crosslinking of chromatin within the plant tissue requires the fixative to penetrate the cells, which is hampered by the plant's waxy cuticle and spongy air-filled mesophyll. In our protocol, efficient penetration of the fixative is achieved by vacuum infiltration of buffer that contains formaldehyde. It is important that the buffer penetrates the plant material completely; after vacuum infiltration the plant material should appear translucent or 'water-soaked'. The buffer volumes used in the presented protocol are suitable for efficiently crosslinking up to 5 grams of plant material. Increasing the amount of material impedes efficient crosslinking.

**Figure 2:**
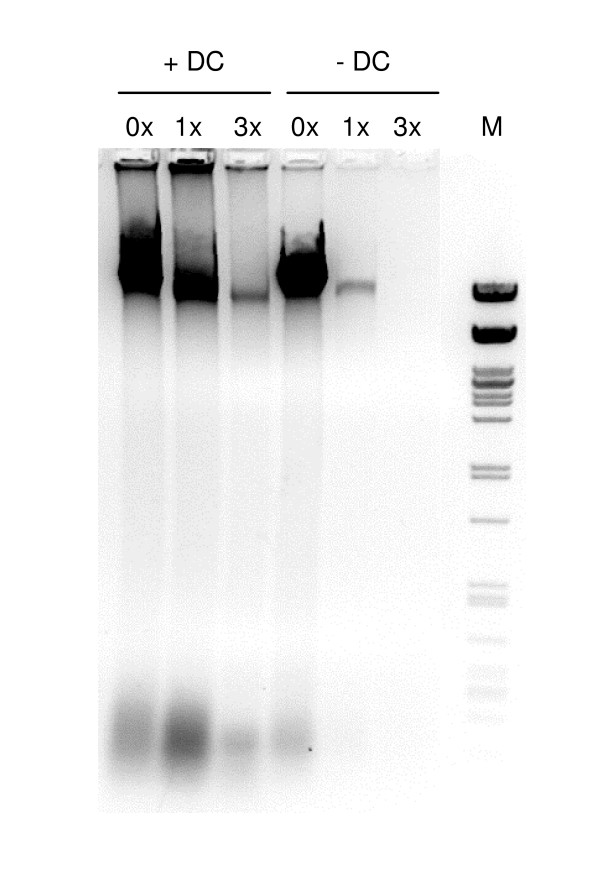
Crosslinking efficiency analysis. Leaf material was crosslinked in buffer containing increasing amounts of formaldehyde. Samples were decrosslinked (+DC) or not (-DC), and DNA isolated using phenol/chloroform extraction followed by ethanol precipitation. While DNA is efficiently isolated from samples that were not crosslinked (lanes indicated with 0×), decrosslinking is required for the isolation of DNA from crosslinked samples (lanes indicated with 1× and 3×). Over-crosslinking strongly decreases DNA isolation efficiency (lanes indicated with 3×). DC, decrosslinking; 0×, 1×, 3×, relative concentration of formaldehyde; M, lambda DNA cut with *Pst*I.

The crosslinking step should be optimized, since too little crosslinking will not sufficiently preserve the chromatin structure, and too much crosslinking will hamper the ChIP procedure [12]. A method to determine the optimal crosslinking conditions is illustrated in Figure 2. Essentially, when the crosslinking is optimal for ChIP, decrosslinking is required to efficiently isolate DNA from the nuclei by phenol-chloroform extraction. The chromatin is over-crosslinked when it is impossible to recover a substantial amount of DNA from the nuclei by decrosslinking. The nuclei are under-crosslinked when most of the DNA can be recovered without decrosslinking. Crosslinked material can be stored for several months at -80°C after freezing in liquid nitrogen.

#### Shearing of chromatin

The resolution obtained by the ChIP procedure is determined by the size of the chromatin fragments used as input material. Two methods are commonly used to fragment chromatin, sonication (hydrodynamic shearing) and micrococcal nuclease (MNase) digestion. Both methods can show preferential fragmentation of certain chromosomal regions [13,14]. When using formaldehyde crosslinking, sonication is the preferred method, as crosslinking restricts the access of MNase to chromatin [15].

Optimal fragmentation can be achieved by testing various sonication conditions on chromatin, followed by DNA isolation and estimating the sonication efficiency by gel electrophoresis. Ideally, the bulk of the chromatin is sonicated to a length between 250 and 750 bp. For efficient fragmentation, sonication at low power, in combination with several pulses, is preferred over sonication at high power and few pulses, but conditions vary with the sonication device used. It is important to keep the chromatin sample cooled on ice during sonication, as heat released by the sonication probe can reverse the crosslinks. The presence of detergent (SDS) in the sonication buffer improves sonication efficiency considerably, but can induce foaming during sonication. Foam makes the chromatin sample unsuitable for ChIP, probably as a result of the surface tension imposed by the foam, which can disrupt protein conformation [16]. Foaming can be prevented by decreasing the sonication power. Sonicated chromatin can be stored at -80°C for at least a few months, but repeated freeze/thaw cycles should be avoided.

### (2) The chromatin immunoprecipitation procedure

#### Choosing antibodies

Antibodies (Ab) are the most important factor for a successful ChIP experiment. It is crucial to choose an antibody carefully, especially when antibodies are raised against non-plant proteins. Papers reporting the use of a specific antibody for ChIP are a good indication of the suitability of that antibody, but one should realize that quality can differ between antibody batches. The successful use of a specific antibody in experiments other than ChIP (i.e. Western blotting, immunocytochemistry) does not automatically mean the antibody is suitable for ChIP; that has to be tested.

Antibodies can be available as polyclonal or monoclonal preparations. Monoclonal antibodies have a high specificity compared to polyclonal sera, but the polyclonal sera may recognize several epitopes of the target, increasing signal levels of low-abundance templates. Be aware that polyclonal serum against multiple modifications (i.e. against hyperacetylated H4) may have an undocumented preference for one modification over the others [17], impeding the biological interpretation of ChIP data.

**Figure 3:**
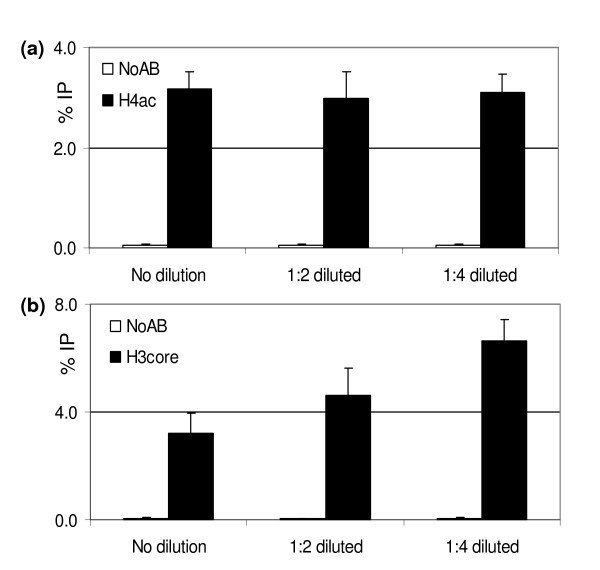
Titration of chromatin relative to a fixed amount of antibody. (a) ChIP titration experiment using an antibody against acetylated H4. Dilution of the chromatin has no effect on the precipitation efficiency. (b) ChIP titration experiment using an antibody against an invariant domain of H3 (H3core). Dilution of the chromatin improves the precipitation efficiency, suggesting that the antibody recognizing the H3core is sensitive to inhibitory factors present in the chromatin sample. ChIP-QPCR was performed as described in this paper. Chromatin was isolated from leaves of 2 week-old plants, sonicated and used in 1:1, 1:2 and 1:4 dilutions for ChIP. The input samples are diluted by the same factors as the chromatin samples. Results are represented as %IP, the error bars indicate the standard error. ChIP samples are represented by closed bars, while open bars indicate the signals from the 'no-antibody' serum controls. Each data point represents the average of two different chromatin samples, each analyzed *in duplo*.

Different antibody preparations have distinct properties, which can affect the ChIP results. The affinity for epitopes differs between antibodies, affecting the resulting signal levels. For example, antibodies can differ in their sensitivity towards crosslinks or adjacent modifications (discussed in [18]). In addition, the relation between the availability of epitopes and antibody binding may not be linear. Some antibodies are sensitive to inhibitory factors present in the input chromatin sample, resulting in a decrease in binding efficiency of the antibody when increasing the amount of input. This is exemplified in Figure 3. We tested the effect of changing the amount of input chromatin on ChIP efficiency (the recovery of DNA relative to the amount of input) for two different antibodies. With an antibody against hyperacetylated H4 (Upstate #06–946), the ChIP efficiency is constant over a broad range of chromatin concentrations. When this experiment is performed with an antibody recognizing an invariant domain of H3 (H3core; Abcam #AB1791), dilution of the input chromatin improves the ChIP efficiency. These data show that the relation between ChIP signal and chromatin input depends on the characteristics of the antibody used. To ensure the comparability of results obtained with different input samples, we recommend determining the optimal Ab: chromatin ratio by titration of the amount of input chromatin, and the use of similar amounts of input chromatin when ChIP results are to be compared.

When establishing the ChIP procedure we recommend using an antibody against an invariant domain of histones as a starting point [19,20]. In our hands, such an antibody usually results in a good signal-to-noise ratio. When setting up ChIP to detect histone acetylation, the initial detection can be facilitated by treating plant material with butyrate or TSA before the isolation of chromatin [7,20]. Such treatments inhibit histone deacetylation, resulting in increased global acetylation levels.

Once the ChIP protocol has been established, it has to be determined which histone modifications will be analyzed. For this purpose, data obtained from immunocytochemistry experiments are a valuable source of information [21,22]. Such experiments should however, be considered as informative but not conclusive for the decision on which histone modification to analyze. Immunocytochemistry experiments provide a global view of the localization of histone modifications at eu- or heterochromatin, while ChIP experiments can have a single-nucleosome resolution [23]. At such high resolution, histone modifications can be observed where they are not expected based on immunolocalization experiments ([20,22]; Haring et al, in preparation). Another factor to keep in mind is that specific chromatin states can be associated with different histone modifications in distinct species. For example, immunolocalization experiments showed the presence of H3K9me2, but not H3K9me3, in heterochromatin of *Arabidopsis*, while H3K9me3, but not H3K9me2, is observed in heterochromatin of mice [21,24,25].

#### Controls for ChIP

To ensure the reliability of ChIP data, two control samples specific for the ChIP experiment should be included: the input sample and the 'no-antibody' (NoAb) control sample (Table 1). Both the input sample and the NoAb control sample provide essential information about the ChIP experiment, and should be analyzed with every primer set used. Additional controls are needed for the QPCR procedure; these will be discussed in the QPCR section.

**Table 1:** Controls for ChIP and QPCR

Controls	Primers sets to test	Purpose
(a) *Controls for ChIP*		
Input Sample	All primer sets	Positive control for presence of chromatin, used to calculate %IP
No Antibody control	All primer sets	Determines background signal level

(b) *Controls for QPCR*		
Calibration line	All primer sets	Positive control for QPCR, used for quantification of QPCR signals
Melting curve	All primer sets	Tests for amplification of the correct fragment
No-template PCR	All primer sets	Negative control for QPCR, indicates primer artifacts

(c) *Controls for data interpretation*		
ChIP control (+)	Control gene primer set	Positive control for ChIP (e.g. *actin *for H3K4me2)
ChIP control (-)	Control gene primer set	Negative control for ChIP (e.g. *actin *for H3K9me2)

The input sample will be indicative for the presence and amount of chromatin used in the ChIP reaction. It is an aliquot taken from the chromatin before preclearing (step 9 in the protocol). The chromatin aliquot is decrosslinked and DNA is isolated. This DNA sample should yield a PCR product with all primer sets used. Besides serving as a positive control, the data derived from the input sample can be used for normalization by the '%IP' method discussed in the section on data normalization.

The NoAb control is a chromatin sample to which non-specific control serum is added instead of a specific antibody (see materials and methods). The NoAb sample is treated the same way as the ChIP samples. The QPCR signals resulting from the NoAb samples indicate the amount of background signal generated by the chromatin preparations and ChIP procedure. Ideally, the washing steps remove non-specifically bound chromatin, resulting in an absence of QPCR signals for the NoAb samples. In reality however, it is not uncommon to find a PCR product for the NoAb control sample. It is very important that the DNA isolated from the NoAb samples is amplified with every primer pair used, as the level of background signal can differ for each primer pair.

#### Optimizing the signal to noise ratio

When setting up ChIP, one of the main problems is a high level of background signal (NoAb control) relative to the level of the signal of interest (ChIP sample). This hampers distinguishing a 'true signal' from the background signal. Therefore, we recommend setting up ChIP using an antibody against an invariant domain of histone H3 [19,20]. In our experiments, such an antibody yields a very clear difference in signal between the ChIP samples and NoAb control. Another helpful tool to discriminate between background and ChIP signal is a positive control sequence for ChIP, as discussed in the 'controls for specific chromatin states' section.

Several options are available to optimize the signal-to-noise ratio. To prevent non-specific binding of chromatin to the protein A/G agarose beads, the beads are blocked with BSA and non-specific blocking DNA before they are used in ChIP assays. To further reduce background, BSA, non-specific serum and unrelated DNA can be added to the pre-incubation step of chromatin with beads (step 9). Options for preventing chromatin from binding to plastic tubes are pre-incubation of the tubes with BSA and unrelated DNA (e.g. salmon sperm DNA), or the use of siliconized tubes. The background can also be reduced by lowering the amount of input chromatin, the amount of protein A/G agarose beads or the amount of antibody, but this may lower signal levels as well.

In case ChIP experiments with an antibody against an invariant domain of histone H3 would result in a low ChIP-QPCR signal relative to the background signal, factors inhibiting the PCR reaction may be involved. We recommend purifying the DNA after the reverse crosslinking (step 17) with a commercial 'PCR purification spin column kit'. In our hands, DNA purification by phenol-chloroform extraction does not sufficiently remove detergents present in the ChIP elution buffer, inhibiting subsequent QPCR reactions.

### (3) Analysis of precipitated material by QPCR

**Figure 4:**
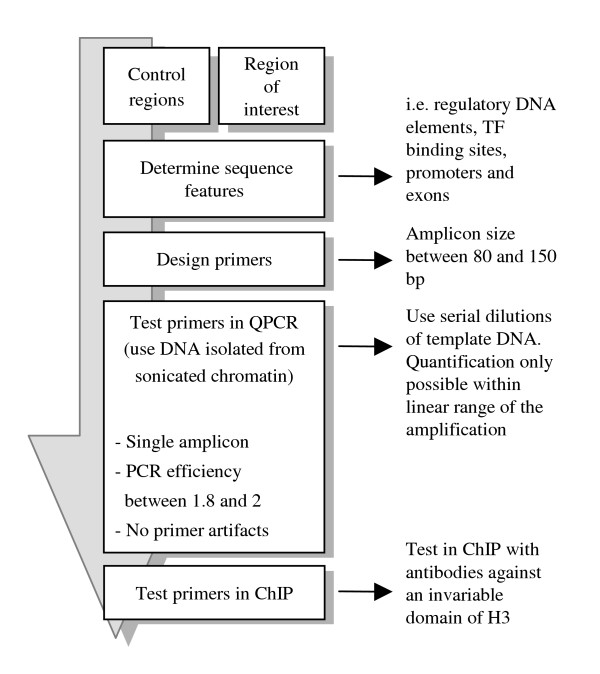
Outline for setting up QPCR. This outline represents setting up the QPCR analysis, as described in the text. Abbreviations: TF: transcription factor.

DNA isolated from the precipitated chromatin has to be analyzed to determine which DNA fragments are present in the precipitate (Figure 1). For the detection of specific DNA fragments, various methods are available, and the chosen method determines to what extent the data can be analyzed quantitatively. Commonly used analysis methods are conventional PCR and quantitative PCR. Alternative methods are microarray analysis and slot blotting. Microarray analysis is useful when studying the genome-wide distribution of histone modifications [26]. Analysis by slot blotting is feasible when dealing with highly repetitive sequences [27,28], but appears not sensitive enough to detect single copy sequences in ChIP samples (M. Haring and M. Stam, unpublished). This paper focuses on quantitative PCR for the detection of immunoprecipitated DNA (see Figure 4 for an outline how to set up QPCR).

Until recently, ChIP precipitates have primarily been analyzed by conventional PCR (e.g. bands on a gel). Such an approach requires lots of testing per individual primer set to ensure that measurements are taken in the linear range of amplification. If this condition is not fulfilled, the resulting data cannot be considered quantitative, impeding data interpretation. For this reason, analyzing ChIP precipitates by quantitative real-time PCR (QPCR) has several advantages over conventional PCR. The QPCR technique does not quantify the amount of PCR product at the end of the PCR reaction, as with conventional PCR band densitometry. Instead, the initial amount of template DNA is calculated from the kinetics of the PCR reaction. During a QPCR run, the accumulation of PCR product is measured every cycle. The number of cycles needed to reach a certain amount of PCR product – 'Cycle threshold' or Ct value [29]-, and a calibration line (see below), are used to calculate the initial amount of DNA template.

Quantitative PCR analysis for ChIP can be performed using DNA-dye based or probe-based PCR product detection chemistries. The most widely used DNA-dye based QPCR chemistry employs the fluorescent dye SYBRgreen for detection of the amplicon. SYBRgreen is only fluorescent when bound to double-stranded DNA (dsDNA), and the amount of fluorescence is proportional to the amount of dsDNA. SYBRgreen detects a PCR product independent of its DNA sequence, so this chemistry can be used for all primer sets. At the same time, this sequence-independent binding of SYBRgreen requires primer sets to be thoroughly optimized; amplification of non-target DNA fragments and formation of primer dimers will also yield fluorescent signal, hampering measurements of the ChIP-QPCR signal.

When using hybridization probe-based chemistries, one or more fluorescently labeled oligonucleotide probes are designed to anneal to a specific DNA sequence [29]. This adds specificity to the detection of PCR fragments, as both the primers and the probe will have to anneal to template DNA to enable detection of the product.

#### QPCR primer sets

In order to obtain high quality QPCR data, the primer sets used need to meet specific criteria [29,30]. If primer sets are not optimized, this may result in amplification artifacts and/or an inaccurate quantification. Primer sets can be tested by performing QPCR on serial dilutions of template DNA isolated from crosslinked, sonicated chromatin. The efficiency of the amplification should be as close as possible to two (a 2× dilution of the template should result in a 1 Ct increase), and should remain constant over a wide range of template concentrations. The amplification efficiency can be improved considerably by designing the length of the amplified fragment between 80 and 150 bp. Importantly, the ChIP-QPCR results should be within the linear range of amplification. To verify that the primer sets amplify the correct fragment, sequencing the obtained PCR product is recommended. When using SYBRgreen for detection of the QPCR signal, the amplification of non-target DNA and the formation of primer dimers should be avoided, as the dye does not discriminate between these and target DNA fragments (discussed above).

The sequence regions to be amplified have to be chosen very carefully. Different combinations of histone modifications mark distinct DNA sequence elements, such as regulatory sequences, promoter- or coding sequences. The histone modifications present at such specific DNA elements can be different from those at the flanking chromatin [31]. We therefore advise to design primer sets within a single DNA element, such as a promoter, intron or other regulatory sequence. When the amplified region spans more than one DNA element, the data obtained by ChIP may consist of a mixture of data, hampering the biological interpretation. In conclusion, knowing as many features of the amplified sequences as possible is useful for the design of useful primer sets.

#### Controls for QPCR

A good-quality QPCR analysis can only be performed when taking along the proper controls. These controls are in addition to the earlier discussed input and NoAb controls, which are specific for the ChIP experiment. This section will discuss the required QPCR specific controls: a calibration line, melting curve analysis and the no-template control (Table 1B).

A QPCR calibration line is required for the quantification of ChIP signals, and should be included for every primer set used. A calibration line consists of QPCR reactions performed with multiple dilutions of template DNA that is isolated from sonicated crosslinked chromatin. The calibration line corrects for differences in PCR efficiency between various primer sets, as well as for differences in signal level between multicopy and single copy sequences. Besides enabling quantification, the calibration line functions as a positive control for the QPCR assay, independent of the ChIP samples. The dilution series for the calibration line must be made fresh from a DNA stock every time, as the quality of diluted DNA rapidly decreases. The latter is probably due to binding of DNA to the surface of the reaction tube [32]. Addition of BSA or unrelated DNA may reduce this effect.

When performing QPCR with SYBRgreen, a melting curve analysis can be performed to determine if the correct fragment is amplified. In this extension of the PCR program, the temperature is gradually increased while continuously measuring SYBRgreen fluorescence. PCR products will denature at a temperature specific for their size and sequence, and this will be measured as a loss of fluorescence. Multiple PCR fragments in one reaction, or the presence of primer-artifacts, will result in a step-wise decrease of fluorescence. Optimal primer sets should yield a single denaturation event. Even so, multiple fragments of similar size and/or sequence may yield identical melting curves. The PCR products should therefore be verified by sequencing before using the primers for ChIP-QPCR analysis. The melting curve analysis is not possible when using QPCR chemistries based on fluorescent oligonucleotide probes.

A no-template control, which is commonly included in conventional PCR, is useful for the detection of master mix contamination and primer dimers. The no-template control is distinct from the NoAb control, as the latter usually results in PCR products.

#### (4) Controls for specific chromatin states

Genomic sequences which are known to be associated with specific histone modifications are essential controls for the interpretation of ChIP data (Table 1C). When, in a ChIP experiment, the regions of interest do not show any enrichment of a specific histone modification, the control sequences can indicate if this is due to a failed experiment or if the outcome reflects the actual situation. Ideally, the controls function as a positive and negative control for several antibodies in various tissues.

The development of positive ChIP controls is challenging as chromatin states can not simply be classified as "active" or "silent", many different types of active and silent chromatin exist. These chromatin states are the result of several different molecular networks that can act together at a particular genomic location, each resulting in a distinct set of histone modifications and other chromatin features [33,34]. Identifying a positive control for a specific chromatin state is facilitated by knowledge of the regulatory processes that underlie the presence of the histone modification of interest.

Here, we will discuss controls for two different chromatin states: transcriptional active and repressed chromatin. In our example, the positive control for active chromatin is a negative control for repressed chromatin and *vice versa*.

##### Active chromatin

Importantly, if antibodies are used against histone modifications associated with active chromatin, the control sequence for active chromatin must result in a positive signal in all tissues of interest. A good candidate is a housekeeping gene that is constitutively expressed, such as the *actin *gene, which is frequently used as a positive control for active chromatin [35-39]. Even if the expression level of a control gene varies in different tissues, it may still function as a good control; quantitative changes in expression level do not necessarily reflect changes in histone modifications.

**Figure 5:**
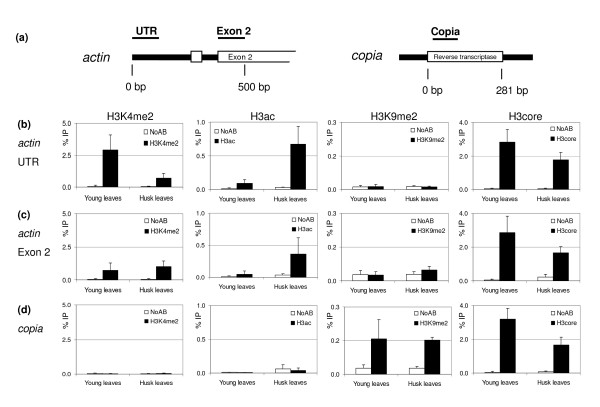
ChIP analysis of control sequences. (a) Location of amplicons used in ChIP-QPCR analysis. The boxed regions indicate part of the coding sequence of maize *actin *1 (genbank #J01238) and the reverse transcriptase sequence of the maize *copia *TY1 type retrotransposon (genbank #AF398212). The amplified sequences are indicated by bars. (b) ChIP-QPCR analysis of the *actin *5' untranslated leader (UTR). (c) ChIP-QPCR analysis of the *actin *exon 2 fragment. (d) ChIP-QPCR analysis of the *copia *sequence (*copia*). ChIP-QPCR is performed as described in this paper. Input chromatin was isolated from leaves of 4 weeks old plants (young leaves) and from husks of 3 months old plants (husk leaves). The ChIP results obtained by 4 independent replicate experiments are represented as percentage of input (%IP), the error bars indicate the standard error. The ChIP signals are represented by closed bars, and open bars indicate the signals from the no-antibody control (NoAb).

We have used maize *actin1 *coding sequences to develop positive controls for active chromatin. Cloning and sequencing of *actin1 *DNA from our maize lines indicated it is a single copy gene and well suited for QPCR analysis. RT-PCR analyses showed it is active in the tissues we have examined (husk and young leaves; data not shown). ChIP was performed as described in this paper, and input chromatin was isolated from leaves of one month old plants (young leaves) and from husks of three months old plants (husk leaves). The precipitates were analyzed with primers specific for two positions on the *actin *gene: the untranslated leader (UTR) and the second exon (exon 2; Figure 5a). We observed positive signals for both the *actin *UTR (Figure 5b) and exon 2 (Figure 5c) when using antibodies against H3K4me2, H3K9ac/K14ac (H3ac), H4ac, but no signals above background with antibodies against H3K9me2 and H3K27me2 (Figure 5 and data not shown). These data show that this sequence can be used as a positive ChIP control for histone modifications associated with active chromatin, and a negative control for histone modifications correlated with repressed chromatin.

Our data in addition demonstrate the importance of carefully selecting the regions to test in ChIP-QPCR (see also the *QPCR primer sets section*). In young leaves, the *actin *exon 2 carries a low level of H3K4me2 (figure 5b), while the *actin *UTR carries a significantly higher level of H3K4me2 (Figure 5c; 95% confidence interval). When performing a ChIP experiment with an antibody against an invariant domain of H3 (H3core), the signals observed for *actin *UTR and exon 2 were comparable, indicating that the difference in H3K4me2 signal levels between UTR and exon 2 is not due to a difference in nucleosome density, but instead reflects a genuine difference in the H3K4me2 levels. This difference is specific for H3K4me2, as the H3ac signal levels are not significantly different between *actin *UTR and exon 2 in young leaves. The observed differences in signal levels of H3K4me2 may reflect differences in the molecular mechanisms acting on the distinct sequence elements of the *actin *gene.

##### Repressed chromatin

To develop a positive control for repressed chromatin, transposon sequences may be considered. Different types of transposons exist, varying in copy number, DNA sequence features and susceptibility to different forms of epigenetic regulation [40,41]. The vast majority of the highly repetitive transposon sequences within plant genomes are truncated [42]. This makes them unfavourable templates for QPCR, because amplification of truncated sequences results in a range of PCR products, impeding correct quantification. For maize, we successfully developed a control for repressed chromatin using the highly conserved TY1 class *copia *LTR retrotransposon reverse transcriptase sequence [43]. The expression of a *copia*-like transposon was shown to be repressed in rice [44], and RT-PCR experiments suggested it is in maize as well (data not shown). We examined the presence of histone modifications at the *copia *sequence (Figure 5a) using the same ChIP precipitates as described above. We observed positive signals with antibodies against H3K9me2 and H3K27me2, without significant differences in ChIP-QPCR signals between young leaves and husk leaves (Figure 5d and data not shown). No signal was obtained in ChIP experiments with antibodies against H3K4me2, H3ac and H4ac (Figure 5d and data not shown). These data show that the *copia *sequence can be used as a positive ChIP control for histone modifications correlated with repressed chromatin, and a negative control for histone modifications correlated with active chromatin.

### (5) Data normalization

The ChIP and QPCR procedure consists of many steps that can influence the final results. Before the obtained data can be interpreted, the variation caused by all these steps must be taken into account. The QPCR data have to be normalized for differences in the amount of input chromatin, precipitation efficiency and variation in the recovery of DNA after the ChIP. Importantly, normalization only serves to correct for this technical variation, it should not affect biological variation. The QPCR data can be normalized by various methods. Since normalization precedes data interpretation, the chosen normalization method can considerably influence the final conclusions. In a high quality ChIP experiment, with an optimal signal-to-noise ratio, the normalization method will not affect the biological interpretation, but in practice, experiments are often not optimal. This makes the choice for the appropriate normalization method essential for correct data interpretation.

The normalization step is often an underestimated part of ChIP experiments. In the literature there is no consensus on how to normalize ChIP-QPCR data, and as a result numerous different normalization methods are being used. Although the perfect normalization method does not exist, some are clearly more prone to result in incorrect data interpretation than others.

The currently used normalization methods for ChIP analysis are background subtraction [45], percent of input (%IP [28]), fold enrichment [46], normalization relative to a control sequence [47] and normalization relative to nucleosome density [48]. The various methods will be discussed here, along with their most important advantages and disadvantages (Table 2). If the data needed for multiple normalization methods are available, it is possible to assess the influence of the various methods on the interpretation of the data. This enables choosing the most applicable method.

**Table 2:** ChIP-QPCR normalization strategies

Normalization strategy	Normalization method	Points of interest
No normalization	ChIP data is not normalized, equal amounts of input chromatin are used in different experiments	Differences in chromatin purity may cause differences in ChIP signal
Background subtraction	The no-antibody signal is subtracted from the ChIP signal	Background signal levels in the NoAb control samples may be different from those in the ChIP samples, depending on the used primer set, chromatin purity and sample handling. The normalization will be strongly influenced by fluctuations in the background signal
Fold enrichment	Normalization to background signal	See *Background subtraction*
% of input	Normalization to the amount of input chromatin	Differences in sample handling between input and ChIP samples affect the normalization
Relative to control genes	Normalization to the ChIP signal obtained at a control sequence	Control sequences need to be developed. Control ChIP signals may differ between different tissues and developmental stages
Relative to nucleosome density	Normalization to the ChIP signal obtained with an antibody for unmodified histone protein	ChIP signals obtained with different antibodies are difficult to compare quantitatively. Regions with low nucleosome density can yield incorrect normalization

#### No normalization

With this method, the absolute signals derived from ChIP-QPCR are not normalized. When using this method, it is assumed that the signals between different samples can be compared without normalization. This is only possible when the amount of input chromatin is very carefully quantified, the quality of the isolated chromatin is highly reproducible and washing and purification steps are performed with absolute accuracy. The advantage of this method is that it requires a minimum of PCR reactions per experiment. The disadvantage lies in the fact that, to be able to compare the results obtained with different samples, equal amounts of chromatin should be used in the ChIP procedure.

#### Background subtraction

With this method the background signals, as measured with the NoAb control, are subtracted from the signals obtained from the ChIP samples. It is assumed that by subtraction of the background signals only the true enrichment is shown. The major shortcoming of this method is the fact that the levels of background signal in the NoAb control samples may be different from those in the ChIP samples. These potential differences in background signal levels can be caused by variation in serum components and by differences in sample handling between NoAb controls and ChIP samples. The level of background QPCR signal can in addition differ between different chromatin samples, primer pairs or separate ChIP experiments. Especially if the signal obtained with particular antibodies is only moderately higher than the background, subtraction will influence data interpretation and should therefore not be done. When the background is very low, subtraction does not influence data interpretation. Nevertheless, the NoAb sample is an essential control for the interpretation of ChIP signals, as it indicates the background signal level for the various primers sets in the different samples. We therefore propose to show the ChIP signals, without background subtraction, side by side with the background signals in all cases.

#### Fold enrichment

This normalization method is also called 'signal over background' or 'relative to the no-antibody control'. With this method, the ChIP signals are divided by the NoAb signals, representing the ChIP signal as the fold increase in signal relative to the background signal. The assumption of this method is that the level of background signal is reproducible between different primer sets, samples and replicate experiments. We strongly recommend not to use this method, because the background signal levels do vary between primer sets, samples and experiments (discussed above). The main disadvantage of this method can best be illustrated with data from a hypothetical ChIP experiment. A ChIP sample that is analyzed with two different primer sets results in a signal of 1 for both primer sets. The background signal (as measured in the NoAb control) is 0.01 for one primer set, and 0.001 for the other. These are realistic values since in our experiments we regularly observe a difference in NoAb control signal level of 10 fold or more between different primer sets. When the 'fold enrichment' normalization method is used for this data set, the normalized signal for the first primer set would be 1/0.01 = 100 and for the second 1/0.001 = 1000. The normalized data now suggest a 10 fold difference in signal level between the two different sequences, solely as a result of the NoAb signal levels. In reality there may be no difference at all. Clearly, the use of this normalization method can result in a random over- or under-representation of the ChIP data.

#### % of input

In this method, the QPCR signals derived from the ChIP samples are divided by the QPCR signals derived from the input sample taken early during the ChIP procedure (Figure 1). It is assumed that the obtained ChIP and NoAb signal levels are directly related to the amount of input chromatin. The main disadvantage of this method is caused by differences in handling between the input and ChIP samples; the input sample is taken from the chromatin solution very early in the ChIP procedure and processed separately from the ChIP samples. Another drawback is that, when comparing different samples, this method does not correct for differences in chromatin contaminations that inhibit antibody-antigen interactions. When the %IP method is used, one should take care that the normalization only corrects for technical variation and does not mask biologically important variation.

#### Relative to control sequences

With this normalization method, the QPCR signal derived from the sequence of interest is divided by the signal derived from the positive sequence control(s), assuming that the chromatin structure at the control sequence does not differ between samples, and that consequently all signal variation is of technical origin. The advantage of this method is that the same ChIP samples are used to analyze sequences of interest and control sequences, eliminating variation caused by sample handling. The disadvantage of this method is that the development and characterization of positive control sequences for all histone modifications of interest can be laborious. This method requires consistent positive QPCR signals for the positive control sequence with all antibodies used. When using probe-based QPCR chemistries, multicolour multiplex QPCR can be used to measure the region of interest and the control sequence in the same tube. Importantly, when control sequences are used to correct for differences in immunoprecipitation efficiency between various tissues, thorough testing of the control sequences is required to determine if all tissues yield identical results.

#### Relative to nucleosome density

The ChIP signals can also be normalized relative to nucleosome density. In that case, the ChIP-QPCR signals obtained with a specific antibody are divided by the signal obtained with an antibody against an invariant domain of a histone, for example histone H3. This normalization method corrects for differences in ChIP signals that are caused by differences in the density of nucleosomes, rather than by changes in histone modification levels.

Histone modifications can only be detected at a specific DNA sequence region if this region is also wrapped into nucleosomes. For example, a relatively low H3K4me2 signal at the transcription start site of a gene, when compared to the flanking regions, can be due to a low nucleosome density rather than a decreased presence of the modification as such [31].

A major disadvantage of this method is that it is very complicated to quantitatively compare ChIP-QPCR signal levels obtained by two different antisera. As discussed in the *Choosing antibodies *section, every antibody preparation has different epitope binding kinetics that are only linear in a specific range of input chromatin. In addition, the relation between epitope concentration and resulting ChIP-QPCR signal may be different for different antibody preparations. For example, a two-fold change in H3core ChIP-QPCR signal may reflect a different change in epitope availability than a two-fold change in a given histone modification ChIP-QPCR signal. This makes normalization to nucleosome density error prone, as the normalized signal is affected in an unpredictable manner. When this method is used to compare chromatin samples from different sources (e.g. root chromatin with leaf chromatin), the normalized data may in addition reflect differences in chromatin quality instead of nucleosome density. As discussed in the *Fold enrichment *section, at sequence regions that do not yield a clear signal above background in an H3core ChIP-QPCR experiment, normalization relative to nucleosome density will affect the signal levels in unpredictable ways and should therefore not be used. It is important to realize that an H3core ChIP-QPCR experiment can also provide biologically significant data on its own, and should be judged as such before deciding to use it for data normalization.

Before choosing a normalization method, the advantages and drawbacks of each method should be considered. Additionally, the choice depends on the experimental setup and the quality and availability of specific controls. As best practice, we propose to normalize ChIP QPCR data by '%IP' or 'relative to control sequences'. In our hands, both methods can be reliably used with high quality measurements of at least four replicates of a ChIP-QPCR experiment. We propose that ChIP experiments are repeated several times and that the results are presented together with the background signal and standard error. We also propose that ChIP data obtained with antibodies to invariant histone domains, when available, are presented as separate data sets.

## Conclusion

In this paper we provide a robust, optimized ChIP protocol, useful handles for setting up this technique, and strategies on how to further optimize the technique for different plant species and tissues of interest. The use of various types of controls that facilitate the interpretation of ChIP data, and the advantages of QPCR for the analyses of ChIP precipitates are discussed. Good controls and quantitative data allow data normalization and statistical analysis, greatly enhancing data interpretation. Normalization of ChIP data often seems an underestimated step of the ChIP-QPCR procedure. In the literature numerous different normalization methods are used, and this paper provides a discussion on why some methods are more prone to affect data interpretation than others. In addition to the aspects discussed in this paper, there are technical aspects of the ChIP procedure that are difficult to control for experimentally, and which could result in incorrect data interpretation. For example, a two-fold increase in ChIP signal can be interpreted as twice as much of the epitope being present, while it can also be due to masking of the epitope by neighbouring modifications [49,50], fixation artifacts or the quality of the input chromatin (see also the paragraph '*Choosing antibodies*'). These pitfalls are not elaborately discussed in this paper, but they are excellently reviewed in other papers (e.g. [7,18]).

The molecular mechanisms determining the turnover rate of histone modifications can also influence data interpretation [7]. Particular histone modifications can exist as very transient marks. For instance, both histone H3 lysine acetylation [51,52] and specific histone H3 lysine methylation marks [53] can undergo turnover. When a particular histone modification has a high turnover rate, only a subset of the crosslinked nucleosomes will carry that modification, resulting in a lower ChIP signal than when that same modification is continuously present. In other words, a relatively low ChIP signal can be due to a high turnover rate of the modification. Additional experiments, for example the use of inhibitors (e.g. TSA or butyrate) to block the turnover of modifications, can provide insight into the dynamic nature of the histone modifications at the genomic locus of interest [7].

## Methods

### ChIP protocol

This protocol is optimized for maize leaf tissue; some adaptations may be required when this protocol is used for other tissues or species.

#### Plant material and crosslinking

1. Take 1–5 grams of fresh plant material (e.g. leaves) for each sample to be analyzed in the ChIP procedure. Cut the tissue in pieces and transfer them to a 50 ml tube. Do not overfill the tube as this will reduce crosslinking efficiency. Submerge the tissue in 30 ml Isolation buffer A. "Clog" the tube with a few pieces of porous filter material (e.g. polystyrene) to prevent floating of the tissue. Vacuum infiltrate for 10 min in an exicator at room temperature.

2. Remove the filter material. Add 2,5 ml 2 M glycine to each 50 ml falcon tube and mix carefully to quench the crosslinking reaction. Vacuum infiltrate for 5 min at room temperature. Wash the tissue three times with plenty of water, and dry it carefully between paper towels.

#### Nuclei isolation

3. Grind the tissue in liquid nitrogen to a fine, dry powder. This material may be stored at -80°C in pre-cooled tubes, or used immediately.

4. Resuspend the ground and frozen material by adding the powder to a 50 ml tube containing 30 ml ice-cold Isolation buffer B, and mix immediately. Incubate for 15 minutes at 4°C with gentle shaking. Filter the solution through 4 layers of Miracloth (Calbiochem) into a new ice-cold 50 ml tube. Centrifuge the filtrate for 20 minutes at 2880 × g at 4°C.

5. Carefully remove the supernatant and resuspend the pellet in 1 ml ice-cold Isolation buffer C. Transfer the solution to a 1,5 ml microcentrifuge tube. Centrifuge at 12000 × g for 10 minutes at 4°C

6. Remove the supernatant and resuspend the pellet in 300 μl ice-cold Isolation buffer D. Add 1500 μl of ice-cold Isolation buffer D to a new 2 ml microcentrifuge tube. Overlay this layer with the nuclei suspension and centrifuge for 1 hour at 16000 × g at 4°C

#### Chromatin sonication

7. Remove all supernatant and resuspend the pellet in 320 μl ice-cold Nuclei Lysis Buffer. Take a 10 μl aliquot from the nuclei and keep it on ice. This will represent 'unsheared' chromatin. Sonicate the remaining nuclei suspension with pulses of 15 seconds at 3 μm amplitude, as many as needed to obtain an average fragment size between 500 and 1000 bp. Keep the tube cooled in ice water during sonication and cool for 30 seconds in between pulses.

8. Spin 5 minutes at 16000 × g at 4°C to pellet the insoluble fraction. Transfer the clear supernatant, which contains the sonicated chromatin, to a new tube. Keep on ice. Take a 10 μl aliquot from the chromatin solution to check the sonication efficiency. The remaining chromatin can be frozen in liquid nitrogen and stored at -80°C, or used immediately in the next step. Add 140 μl TE, 5 μl 5 M NaCl and 5 μl 20% SDS to the aliquots of 'unsheared chromatin' and 'sonicated chromatin'. Reverse crosslink both samples overnight at 65°C, isolate DNA and estimate the sonication efficiency and chromatin concentration by agarose gel electrophoresis.

#### Chromatin preclearing and input control

9. Add together in a 2 ml tube placed on ice: 300 μl chromatin, 1660 μl ice-cold ChIP Ab incubation buffer and 40 μl washed and blocked protein A Agarose beads. Take a 55 μl aliquot from the chromatin solution (be careful not to take any beads) and store it on ice as the 'input sample' (see step 15 for further handling). Incubate the remaining chromatin sample 1 hour at 4°C with gentle agitation (Rotator at 12 rpm).

#### Immunoprecipitation

10. Centrifuge the chromatin samples for 1 minute at 2300 × g at 4°C to pellet the beads. Take three new 1.5 ml tubes and transfer 550 μl of chromatin solution to each tube. One tube will serve as the 'no-antibody' control (NoAb), the other two will be used for precipitation with antibodies (IP). Add to each tube 430 μl ChIP Ab incubation buffer and 20 μl protein A Agarose beads.

11. Add 1 to 10 μl of antibodies to each of the two IP tubes. The two samples can be used for the same antibody *in duplo*, or for two different antibodies. The amount of antibody to be added has to be determined by titration experiments. Add an equal volume of pre-immune serum or blocking serum to the NoAb control tube. Subsequently, add 20 μl protein A Agarose beads to all three tubes. Incubate the tubes for at least 2.5 hours, or overnight at 4°C with gentle agitation.

12. Centrifuge the tubes for 2 minutes at 200 × g at 4°C. To prevent loss of beads, make sure that all the beads have sunk to the bottom of the tube before proceeding.

13. Wash with the following buffers: 1 × Low Salt Buffer, 1 × High Salt buffer, 1 × LiCl buffer, and 2 × with TE buffer. Wash with 1 ml buffer for 10 minutes at 4°C with gentle agitation. After each wash, spin the beads for 1 minute at 200 × g at 4°C, and discard the supernatant.

14. Remove all buffer, but take care not to lose any beads. Add 250 μl ChIP Elution buffer to the pelleted beads to elute the immune complexes. Vortex briefly and incubate at 65°C for 15 minutes with gentle agitation. Spin the beads for 2 minutes at 3500 × g at room temperature. Transfer the supernatant into a new tube, and repeat the elution step. Combine the second eluate, together with the beads, with the first eluate.

#### Reverse crosslinking

15. Add 20 μl 5 M NaCl to the NoAb tube and two IP tubes. Add 100 μl TE buffer, 6.5 μl 5 M NaCl and 8 μl 20% SDS to the 'input sample' tube (step 9). Reverse crosslink all samples at 65°C overnight.

#### DNA isolation

16. Purify the samples using a commercial spin column kit, and elute the column twice with 41 μl of the supplied elution buffer or TE buffer (end volume will be ~80 μl).

17. Use 2–5 μl for a 25 μl PCR reaction. In case the QPCR on the precipitate does not work efficiently, try out if more, but also if less of the DNA sample solves the PCR problems. It can occur that inhibiting factors are present in the DNA sample.

#### ChIP buffers and chemicals

All buffers must be made fresh and kept on ice, unless indicated otherwise.

##### Isolation buffer A

10 mM Tris pH 8.0, 400 mM Sucrose, 10 mM Na-butyrate, 3% w/v formaldehyde, 0.1 mM PMSF, 5 mM Beta-Mercaptoethanol.

##### Isolation buffer B

10 mM Tris pH 8.0, 400 mM Sucrose, 10 mM Na-butyrate, 0.1 mM PMSF, 5 mM Beta-Mercaptoethanol, proteinase inhibitors 1 μg/ml each (see below).

##### Isolation buffer C

10 mM Tris pH 8.0, 250 mM Sucrose, 10 mM Na-butyrate, 10 mM MgCl_2_, 1% v/v Triton ×-100, 0.1 mM PMSF, 5 mM Beta-Mercaptoethanol, proteinase inhibitors 1 μg/ml each (see below).

##### Isolation buffer D

10 mM Tris pH 8.0, 1.7 M Sucrose, 10 mM Na-butyrate, 2 mM MgCl_2_, 0.15% v/v Triton ×-100, 0.1 mM PMSF, 5 mM Beta-Mercaptoethanol, proteinase inhibitors 1 μg/ml each (see below).

##### Nuclei Lysis buffer

50 mM Tris pH 8.0, 10 mM EDTA, 0.4% w/v SDS, 0.1 mM PMSF, proteinase inhibitors 1 μg/ml (see below).

##### ChIP Ab incubation buffer

50 mM Tris pH 8.0, 1 mM EDTA, 0.1% v/v Triton ×-100, 150 mM NaCl, 10 μg/ml BSA.

##### Low salt wash buffer

20 mM Tris pH 8.0, 2 mM EDTA, 0.1% w/v SDS, 1% v/v Triton ×-100, 150 mM NaCl

##### High salt wash buffer

20 mM Tris pH 8.0, 2 mM EDTA, 0.1% w/v SDS, 1% v/v Triton ×-100, 500 mM NaCl.

##### LiCl wash buffer

20 mM Tris pH 8.0, 1 mM EDTA, 1% v/v NP-40, 1% w/v Na-deoxycholate, 250 mM LiCl.

##### TE buffer

10 mM Tris pH 8.0, 1 mM EDTA.

##### ChIP Elution buffer

1% w/v SDS, 0.1 M Na-HCO3. Keep at room temperature.

##### Proteinase inhibitors

Aprotinin, Leupeptin and Pepstatin. Aprotinin inhibits serine proteases; Leupeptin inhibits serine and thiol proteases; Pepstatin inhibits aspartic proteases. Make 1 mg/ml each in MilliQ, aliquot and store at -20°C. It is also possible to use commercially available proteinase inhibitor tablets.

##### Blocked protein A agarose beads

Take 1 ml commercially available pre-swollen agarose beads with crosslinked recombinant Protein A/G (provided as a 50% v/v suspension). Remove the supernatant and wash three times in a total volume of 1 ml with TE buffer. After each wash step, allow the beads to settle and remove the supernatant. Resuspend the beads in a total volume of 1 ml TE buffer with BSA and sonicated salmon sperm DNA to a final concentration of 10 μg/ml each. Add Na-azide to a final concentration of 0.05% v/v. Incubate overnight at +4°C with gentle rotation (12 RPM). Store at +4°C.

#### Antibodies

Immunoprecipitation was carried out using antibodies against H3K4me2 (Upstate #07–030, Upstate USA, Chicago, USA), H3K9ac/K14ac (Upstate #06–599), H3K9me2 (Upstate #07–441), histone H3 (Abcam #ab1791, Abcam plc, Cambridge, UK) and Hyperacetylated histone H4 (Upstate #06–946) and non-specific control serum (Sigma #R9133, Sigma Aldrich, St. Louis, USA) according to the protocols described in this paper. This paper uses the nomenclature for modified histones as proposed by the Epigenome Network [54].

### QPCR analysis

Quantitative (real time) PCR was performed using the Platinum SYBR Green QPCR supermix-UDG kit (Invitrogen #11733–046, Invitrogen, Carlsbad, USA) in a 25 μl QPCR reaction according to the manufacturer's protocols. The samples were amplified using an Applied Biosystems 7500 Real-Time PCR System (Applied Biosystems, Foster City, USA), and quantified with a calibration line made with DNA isolated from crosslinked, sonicated chromatin. With all experiments, no-template controls, NoAb controls and input samples were taken along for every primer set used. Two primer sets were developed for the *actin1 *gene (genbank #J01238), and one primer set on the reverse transcriptase sequence of *copia *(genbank #AF398212; Fw: forward, Rev: reverse): *actin *UTR: Fw TTTAAGGCTGCTGTACTGCTGTAGA; Rev: CACTTTCTGCTCATGGTTTAAGG (primer set amplifies basepair 10–129 of #J01238); *actin *exon2: Fw: GATGATGCGCCAAGAGCTG; Rev: GCCTCATCACCTACGTAGGCAT (amplifies bp 377–480 of #J01238); *copia*: Fw: CGATGTGAAGACAGCATTCCT; Rev: CTCAAGTGACATCCCATGTGT (amplifies bp 6–112 of #AF398212)

### Plant stocks

Maize stocks used for ChIP analysis (inbred W23 and K55 background) are obtained from the Chandler laboratory (Plant Sciences Department, University of Arizona, Tucson, Arizona, USA), and Cultivar Montello from AGASaat GmbH, Neukirchen, Germany.

## Competing interests

The author(s) declare that they have no competing interests.

## Authors' contributions

MH adapted and optimized the ChIP protocol for efficient and routine use on plant material, designed and performed the experiments shown in Figure 5, and drafted the manuscript. SO adapted and optimized the ChIP protocol for efficient and routine use on plant material and designed and performed the experiments shown in Figure 2. TD contributed to the optimization of the experiments shown in Figure 5. IH designed and performed the experiment shown in Figure 3. CP contributed to the design and interpretation of the data shown in Figure 2 and 3, and participated in the preparation of the manuscript. MS coordinated the project, participated in its design and drafted the manuscript. All authors read and approved the final manuscript.
